# Pulse rate variability is not the same as heart rate variability: findings from a large, diverse clinical population study

**DOI:** 10.3389/fphys.2025.1630032

**Published:** 2025-07-30

**Authors:** Allen B. Kantrowitz, Kfir Ben-David, Michael Morris, Harrison L. Wittels, Michael J. Wishon, Samantha M. McDonald, Eric J. Renaghan, Luis A. Feigenbaum, S. Howard Wittels

**Affiliations:** ^1^ Department of Neurosurgery, Mount Sinai Medical Center, Miami Beach, FL, United States; ^2^ Department of Surgery, Mount Sinai Medical Center, Miami Beach, FL, United States; ^3^ Department of General Surgery, College of Medicine, University of Florida, Gainsville, FL, United States; ^4^ Department of Pulmonology, Brooke Army Medical Center, Fort Sam Houston, TX, United States; ^5^ San Antonio Uniformed Services Health Education Consortium, Uniformed Services University, San Antonio, TX, United States; ^6^ Tiger Tech Solutions, Inc., Miami, FL, United States; ^7^ Science, Technology and Research Organization, Miami, FL, United States; ^8^ School of Kinesiology and Recreation, Illinois State University, Normal, IL, United States; ^9^ Department of Athletics, Sports Science, University of Miami, Miami, FL, United States; ^10^ Department of Physical Therapy, Miller School of Medicine, University of Miami, Miami, FL, United States; ^11^ Department of Anesthesiology, Mount Sinai Medical Center, Miami, FL, United States; ^12^ Department of Anesthesiology, Wertheim School of Medicine, Florida International University, Miami, FL, United States; ^13^ Miami Beach Anesthesiology Associates, Miami, FL, United States

**Keywords:** cardiovascular, heart rate variability, pulse rate variability, pulse wave, wearable, technology, autonomic nervous system, electrocardiogram

## Abstract

**Introduction:**

Scientists and consumer products are increasingly employing light-based photoplethysmography (PPG) instead of electrocardiography (ECG) assuming it accurately quantifies heart rate variability (HRV). Recent studies, however, have demonstrated that pulse rate variability (PRV) derived from PPG is not equivalent to HRV-derived from ECG. This study investigated the agreement between PPG-PRV and ECG-HRV in a beat-to-beat analysis in 931 adults recruited from a tertiary academic medical center in the southeastern United States.

**Methods:**

Participants wore two (chest and bicep) Warfighter Monitor™ devices (Tiger Tech Solutions, Inc.). Heart rate (HR), pulse rate (PR) and three time-domain indices for PPG-PRV and ECG-HRV were measured. ECG-derived RR and noise-filtered NN intervals were extracted to compute HR, SDNN (standard deviation of NN intervals), rMSSD (root mean square of successive differences), and pNN50 (percentage of successive NN intervals differing by >50 ms). PPG-derived pulse-wave peaks were detected to calculate corresponding PR/PRV metrics. Pearson correlation, Bland–Altman, and one-way ANOVA analyses assessed linear association, bias, and mean differences across select chronic diseases.

**Results:**

Significant disagreement and differences were observed between ECG-HRV and PPG-PRV (p < 0.001 for all). For rMSSD: *cardiovascular:* 3.04 ms, 95% CI: 1.33, 4.75, *endocrine*: 2.85 ms, 95% CI: 0.52, 5.18, and *neurological:* 4.39 ms, 95% CI: 1.39, 7.39). For SDNN: *cardiovascular:* 8.50 ms, 95% CI: 5.25, 11.74, *endocrine:* 8.43 ms, 95% CI: 3.97, 12.90, *neurological:* 11.84 ms, 95% CI: 6.02, 17.67, and *respiratory:* 7.23 ms, 95% CI: 1.83, 12.62). For pNN50: *cardiovascular:* 2.48%, 95% CI: 1.67, 3.3, *endocrine*: 2.21% 95% CI: 1.12, 3.29, *neurological:* 2.91%, 95% CI: 1.25, 4.32, and *respiratory:* 1.46%, 95% CI: 0.15, 2.77).

**Discussion:**

PPG-PRV is a poor surrogate for ECG- HRV as it significantly underestimated SDNN, rMSSD, and pNN50 across select chronic diseases. Given the widespread use of PPG-based devices and ubiquitous, incorrect assumption that PRV accurately reflects HRV, researchers, clinicians, and manufacturers must clearly distinguish between PRV and HRV in studies and product claims.

## 1 Introduction

Millions of people use wearable smart devices to monitor their health and well-being, with the aim of tracking physiological signals as indicators of autonomic nervous system (ANS) activity. Fluctuations in ANS function provide critical insights into an individual’s risk for chronic diseases such as cardiovascular conditions (Hillebrand et al., 2013), cancer (Hu et al., 2018; Wu et al., 2021), and type 2 diabetes (Trivedi et al., 2019). Artificial intelligence (AI) may soon leverage these health data to assist clinicians in non-invasively detecting chronic diseases (Secinaro et al., 2021). However, the accuracy of health tracking and AI-driven models depends on properly labeling and understanding these metrics. A major concern is the use of photoplethysmography (PPG) to measure “heart rate variability (HRV)” in wearable devices and academic studies, despite its fundamental differences from the gold-standard electrocardiogram (ECG) (Mejía-Mejía et al., 2020a; Mejía-Mejía et al., 2020b). This discrepancy has provoked considerable scientific scrutiny over PPG accuracy (Hoog Antink et al., 2021). Given these concerns, the scientific community has an ethical responsibility to rigorously investigate this and similar issues to prevent widespread misinformation, potential false claims, and barriers to life-saving interventions.

The ANS regulates many vital physiological processes and is highly sensitive to internal (e.g., catecholamines, hormones) and external (e.g., temperature, caffeine, exercise) stimuli (Benarroch, 2020). The downstream responses of these processes communicate the changes occurring in ANS activity, indicating the status (normal vs. abnormal) of ANS function. The cardiac cycle, specifically, instantaneously reflects fluctuations in ANS activity as its electrical impulses are highly sensitive to the catecholamines released by the sympathetic and parasympathetic branches of the ANS. Thus, any variations in cardiac activity, i.e., HRV, serve as a proxy measure of changes in ANS activity and function. Importantly, cardiac activity is easily and non-invasively measurable, most precisely, with ECG since it directly measures the electrical activity of the cardiac cycle (Sattar and Chhabra, 2025). The ECG captures the de- and repolarization of electrical signals clearly delineating the systolic and diastolic phases of the cardiac cycle, represented by the QRS complex. Importantly, the ECG precisely measures the minute variations occurring between each cardiac cycle, specifically the R-R interval, that is HRV, in both time and frequency domains.

The traditional placement of ECG leads on the chest and the difficulty associated with wearing them has driven the exploration of alternative methods, such as PPG, for measuring “HRV” as it is simpler to wear on the wrist/finger/etc. Regardless of placement, PPG fundamentally differs from ECG, although they both track cardiac activity, PPG measures fluid dynamics whereas ECG measures electrical signals. Specifically, PPG uses optical technology to measure dynamic fluctuations in blood volume within the field of view of an optical sensor (Allen, 2007; Njoum and Kyriacou, 2017). Cardiac systole and diastole affect blood volume thereby altering the intensity of light penetrating the microvasculature (Castaneda et al., 2018; Nitzan and Ovadia-Blechman, 2022). Blood flows smoothly throughout the arterial vasculature following the systolic and diastolic phases of the cardiac cycle. In the microvasculature, the microscopic blood vessels within the field of view of an optical sensor exhibit biphasic changes in diameter, altering the amount of light absorbed. The PPG signal is filtered by the physical shape of the blood vessels, acting as a structural low-pass filter. This process naturally generates smooth, pulsatile waves with rounded peaks (Castaneda et al., 2018). The number of pulsatile waves over time represents a pulse rate (PR). The fundamental differences between ECG and PPG signals create significant challenges. Unlike the ECG, where the QRS complex clearly defines fiducial points such as the precise timing of the “R peak,” PPG signals exhibit a smooth, wave-like pattern. This rounded shape of the PPG wave makes it difficult to pinpoint the exact peak, leading to discrepancies between HRV and PRV (Mejía-Mejía et al., 2020a; Mejía-Mejía et al., 2020b). Additionally, this discrepancy affects the quantification of amplitudes and diminishes the subtle variations crucial to HRV analysis. Essentially, the minute fluctuations being tracked by HRV are filtered out in PRV. Thus, this stark difference between the PPG and ECG methodologies and their derivatives casts significant doubt on whether PPG-derived PRV can measure HRV with a scientifically acceptable level of accuracy.

Despite these significant physiological differences, PPG and its derivative, PRV, continue to be used as surrogates for ECG and HRV. Previous studies have varied widely in methodology, including differences in measurement sites, small and less diverse sample sizes, and analytical approaches (Schäfer and Vagedes, 2013; Yuda et al., 2020b; Farhan et al., 2024). These inconsistencies may have contributed to ambiguous conclusions about the limitations of PRV and its potential downstream consequences. Thus, the current study aims to address the weaknesses of other studies by employing a large-scale study using *one* wearable device equipped with *both* ECG and PPG capabilities in a sample of 931 United States adults exhibiting diverse demographic and health profiles. We hypothesized that PPG-derived PRV would exhibit poor agreement with standard HRV metrics and consistently across major health conditions, clearly demonstrating that PRV is as an invalid surrogate for HRV.

## 2 Materials and methods

### 2.1 Study design

This is a cross-sectional study among a diverse patient population recruited from a single U.S. medical institution. ECG and PPG were measured using a non-invasive armband monitor prior to each patient’s doctor’s appointment.

#### 2.1.1 Recruitment and study sample

Patients with any scheduled surgical or non-surgical procedures (e.g., colonoscopy, Pap smear, biopsy) were recruited from a tertiary care academic medical center in the southeastern region of the United States from May 20, 2024 to September 23, 2024. Patient health information was blindly extracted from medical records by study personnel 1 week following the patient’s HRV measurement. In total, we recruited and measured HRV and PRV on 931 patients. The demographic profile of the study sample was 53.4% male, 37.4% non-Hispanic White, 53.7% Hispanic or Latino, 6.9% Black or African American and ranging from 17 to 99 years of age. For the health profile, 47.8% of patients were classified as obese (body mass index ≥30 kg/m^2^. A significant proportion of patients exhibited at least one health condition like cardiovascular (61.2%), respiratory (31.5%), cancer (20.3%), endocrine (43.4%), neurological (32.5%), etc. Table 1 presents the prevalence the different types of health conditions. All study protocols and procedures followed the principles stated in the Declaration of Helsinki and were approved by the Mount Sinai Medical Center Institutional Review Board. Patients were fully informed of the study details and voluntarily provided consent. Health markers were curated independently by the doctors, while the HRV/PRV markers were calculated separately, the two were then combined an analyzed in a double-blind manner.

**TABLE 1:** Demographic and health profiles of total study sample.

Sample characteristics	Mean (SD)	Min, Max
N = 931		
Demographic profile		
Sex (% male)	53.49%	-----
Race (%)		
African American or Black	6.98%	-----
Asian	1.39%	-----
Caucasian or White	37.49%	-----
Other	0.21%	-----
Ethnicity (%)		
Hispanic or Latino	53.7%	-----
Age (years)	60.91 (16.17)	17.0, 97.0
Health profile		
Height (m)	1.69 (9.88)	1.24, 2.05
Weight (kg)	78.62 (18.22)	39.92, 157.40
Body Mass Index (kg/m^2^)	27.32 (5.69)	14.29, 52.72
No. of Comorbidities		
0	10.09%	-----
1	17.19%	-----
2	24.70%	-----
3	23.73%	-----
≥4	24.27%	-----
Heart Rate (bpm)		
ECG _chest_	72.26 (14.30)	-----
ECG _bicep_	72.30 (14.40)	-----
PPG _bicep_	72.49 (14.55)	-----
rMSSD (ms)		
ECG _chest_	43.10 (13.40)	-----
ECG _bicep_	43.14 (13.27)	-----
PPG _bicep_	37.49 (11.66)^a^ ^,^ ^b^	-----
SDNN (ms)		
ECG _chest_	77.23 (26.40)	-----
ECG _bicep_	77.21 (26.88)	-----
PPG _bicep_	64.10 (21.91)^a^ ^,^ ^b^	-----
pNN50 (ms)		
ECG _chest_	22.10 (6.39)	-----
ECG _bicep_	22.08 (6.06)	-----
PPG _bicep_	18.12 (5.24)^a^ ^,^ ^b^	-----

^a^ Denotes statistically significance (p < 0.00001) between *PPG*_*bicep*_ and *ECG*_*chest.*_

^b^ Denotes statistically significance (p < 0.00001) between *PPG*_*bicep*_ and *ECG*_*bicep.*_

See Supplementary Table S1 for complete list of comorbidities.

#### 2.1.2 ANS function via heart rate and pulse rate variability

Heart rate (HR), pulse rate (PR) and three, time-domain metrics for HRV and PRV were measured using an armband monitor (Warfighter Monitor™ [WFM], Tiger Tech Solutions, Miami, FL) equipped with electrocardiographic and photoplethysmographic technology. The WFM was previously validated in similar subpopulations (Peck et al., 2021; Peck et al., 2023; Renaghan et al., 2023; Temme et al., 2023). Patients were fitted with WFM on the upper left arm around the widest posterior aspect of the biceps muscle and secured with an elastic strap. Another WFM device was placed on the patient’s chest and simultaneously measured HR and HRV. Patients were instructed to remain seated in an upright position, nearly motionless and breathing at their normal rate for 5–7 min (Ajdaraga and Gusev, 2017).

#### 2.1.3 Heart rate variability

HRV metrics were calculated using the changes in the inter-beat intervals. RR intervals were the time between R waves on consecutive QRS complexes and NN intervals were noise-free RR intervals. R peaks were detected utilizing a modified Pan-Tompkins algorithm (Elgendi, 2013). Noise-free RR intervals were validated using established signal quality indices (SQI) (Rahman et al., 2022). From this data, three separate time-domain indices were derived including SDNN (standard deviation of the NN interval), rMSSD (the root mean square of successive differences between NN intervals), and the percentage of time in which the change in successive NN intervals exceeds 50 ms within a given measurement (pNN50). These HRV time-domain indices are well known to reflect parasympathetic and sympathetic autonomic output (Task Force of the European Society of Cardiology and North American Society of Pacing and Electrophysiology, 1996; Ernst, 2017; Shaffer and Ginsberg, 2017). We utilized an ECG sampling rate of 100 Hz which provides sufficient bandwidth to detect QRS peaks bandpass filtered between 8 and 15 Hz. Importantly, the WFM previously demonstrated strong correlations with a standard 2-lead chest ECG (*R* ^2^ = 0.95) for measuring the frequency and variations in R-R intervals (Peck et al., 2021).

#### 2.1.4 Pulse rate variability

PPG technology, housed in the WFM, was used to measure PR and PRV via blood volumetric changes. Using a derivative based algorithm, peaks in PPG-generated pulse waves were detected and defined as the highest amplitude reached for each pulse wave recorded. PR was defined as the frequency of pulse wave peaks detected in a 60 s interval. As in previous studies (Nitzan and Ovadia-Blechman, 2022), pulse wave peaks in the current study were assumed equivalent to the R peak on QRS complex measured on an ECG. Thus, the methods for extracting noise-free “RR” intervals and subsequent indices of PRV were identical with those utilized for HRV described above.

### 2.2 Statistical analysis

The analyses performed evaluated the relationship and agreement between HR/HRV and PR/PRV measured via electrocardiography (at the chest and bicep) and photoplethysmography, respectively. Pearson correlations and Bland-Altman (Bland and Altman, 1986) analyses were performed evaluating the agreement between ECG-Chest vs. ECG-Bicep vs. PPG-Bicep for HR, rMSSD, SDNN and pNN50. Mean differences in HR and HRV estimates between the three measures were compared using an ANOVA. Multiple comparisons were performed using Tukey’s test and adjusted for familywise error. Analyses were also stratified and performed separately for five different categories of chronic diseases including cardiovascular vs. no cardiovascular, endocrine vs. no endocrine, neurological vs. no neurological, respiratory vs. no respiratory and “other” vs. no “other”. Patients in a “no” condition, did not have the respective condition, however, could have presented with other chronic conditions. The *a priori* alpha level was set at ɑ < 0.05. All statistical analyses were performed in MATLAB, version 2021b (MathWorks, Natick, MA, United States).

## 3 Results

**FIGURE 1:**
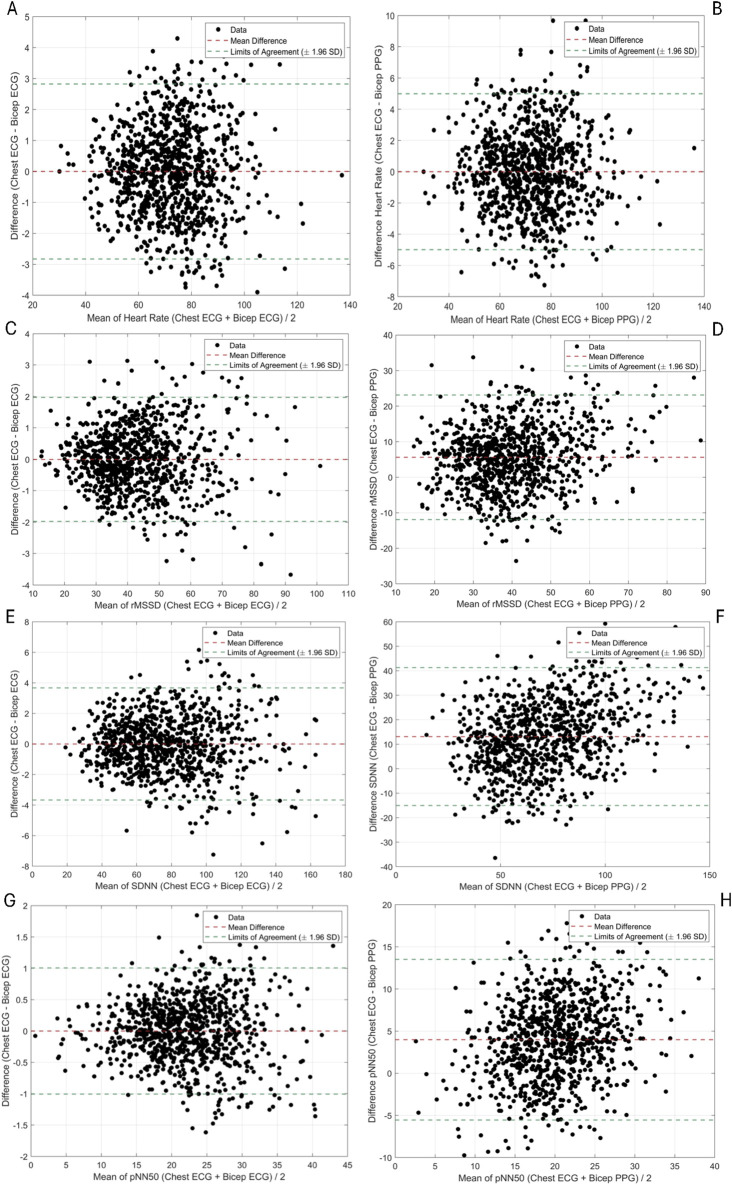
Bland Altman Plots Evaluating Agreement Between ECG and PPG Methods in Measuring HRV Metrics. [**(A)**, top row, left] HR: ECG _chest_ vs. ECG _bicep_ [**(B)**, top row, right] HR: ECG _chest/bicep_ vs. PPG _bicep,_ [**(C)**, 2^nd^ row, left] rMSSD: ECG _chest_ vs. ECG _bicep_, [**(D)**, 2^nd^ row, right] rMSSD: ECG _chest/bicep_ vs. PPG _bicep_, [**(E)**, 3^rd^ row, left] SDNN: ECG _chest_ vs. ECG _bicep_, [**(F)**, 3^rd^ row, right] SDNN: ECG _chest/bicep_ vs. PPG _bicep_, [**(G)**, 4^th^ row, left] pNN50: ECG _chest_ vs. ECG _bicep_ and [**(H)**, 4^th^ row, right] pNN50: ECG _chest/bicep_ vs. PPG _bicep_.

**FIGURE 2:**
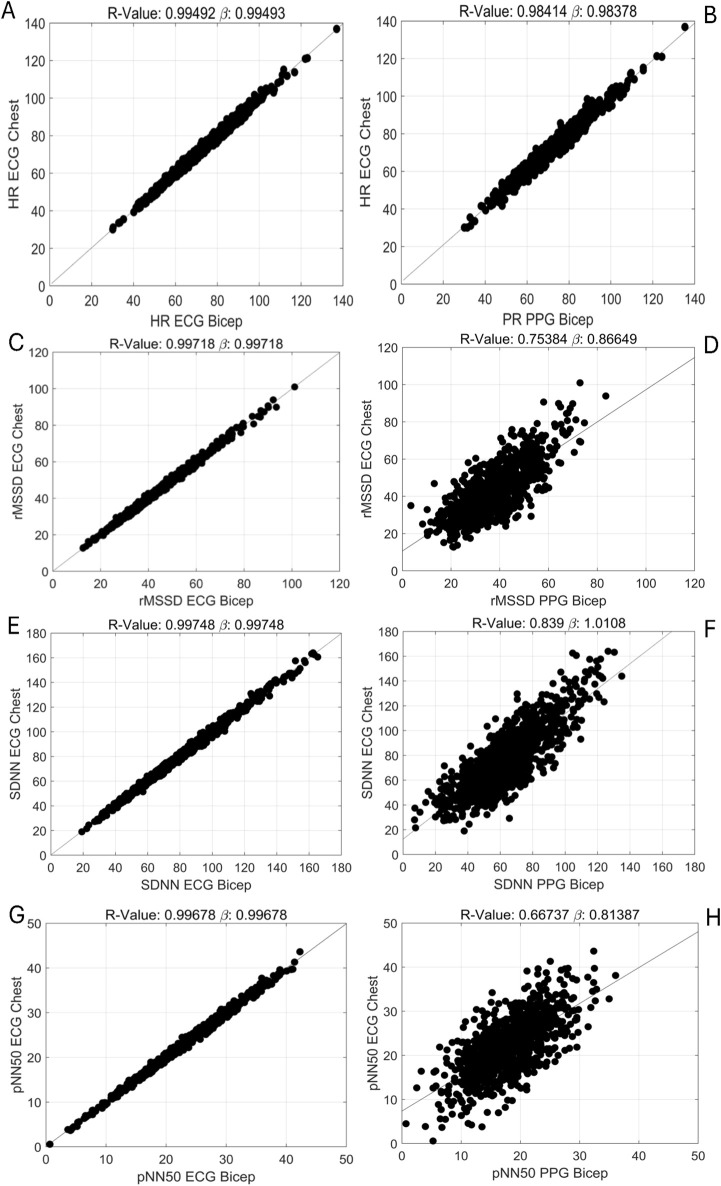
Pearson Correlations Evaluating the Relationships Between ECG and PPG Methods in Measuring HRV Metrics. [**(A)**, top row, left] HR: ECG _chest_ vs. ECG _bicep_ [**(B)**, top row, right] HR: ECG _chest/bicep_ vs. PPG _bicep,_ [**(C)**, 2^nd^ row, left] rMSSD: ECG _chest_ vs. ECG _bicep_, [**(D)**, 2^nd^ row, right] rMSSD: ECG _chest/bicep_ vs. PPG _bicep,_ [**(E)**, 3^rd^ row, left] SDNN: ECG _chest_ vs. ECG _bicep,_ [**(F)**, 3^rd^ row, right] SDNN: ECG _chest/bicep_ vs. PPG _bicep_, [**(G)**, 4^th^ row, left] pNN50: ECG _chest_ vs. ECG _bicep_ and [**(H)**, 4^th^ row, right] pNN50: ECG _chest/bicep_ vs. PPG _bicep._

The degrees of agreement between ECG-Chest, ECG-Bicep and PPG-Bicep for HR and HRV using Bland-Altman plots and Pearson correlations are depicted in Figures 1, 2. The estimated mean differences between these measurement methods are in Table 2. For HR, the degree of agreement for the ECG measurement methods and ECG vs. PPG were high, with near-zero mean differences ranging from −0.2 to 0.05 none of which reach statistical significance (see Table 2). In further support, the Pearson correlation coefficients ranged between 0.98 and 0.99, suggesting a strong relationship between ECG-chest, ECG-bicep, and PPG-bicep regarding accurately measuring HR and PR.

**TABLE 2:** Mean differences in HR and HRV between ECG-Bicep and PPG-Bicep methodologies by certain morbidities.

Morbidity^a^	HR (bpm)		rMSSD (ms)		SDNN (ms)		pNN50 (%)	
	Mean (95%CI)	p-value	Mean (95%CI)	p-value	Mean (95%CI)	p-value	Mean (95%CI)	p-value
Cardiovascular								
Yes	0.05 (−1.79, 1.91)	1.00	3.04 (1.33, 4.75)	<0.00001	8.50 (5.25, 11.74)	<0.00001	2.48 (1.67, 3.3)	<0.00001
No	0.04 (−2.29, 2.36)	1.00	9.76 (7.61, 11.9)	<0.00001	20.42 (16.33, 24.50)	<0.00001	6.27 (5.24, 7.29)	<0.00001
Endocrine								
Yes	−0.21 (−2.73, 2.30)	1.00	2.85 (0.52, 5.18)	0.0051	8.43 (3.97, 12.90)	<0.00001	2.21 (1.12, 3.29)	<0.00001
No	−0.17 (−2.38, 2.02)	1.00	7.79 (5.75, 9.83)	<0.00001	16.71 (12.80, 20.62)	<0.00001	5.29 (4.33, 3.29)	<0.00001
Neurological								
Yes	−0.20 (−3.53, 3.12)	1.00	4.39 (1.39, 7.39)	0.00027	11.84 (6.02, 17.67)	<0.00001	2.91 (1.25, 4.32)	<0.00001
No	−0.18 (−2.50, 2.13)	1.00	6.25 (4.17, 8.33)	<0.00001	13.73 (9.69, 17.78)	<0.00001	4.45 (3.47, 5.43)	<0.00001
Respiratory								
Yes	−0.22 (−3.23, 2.79)	1.00	2.66 (−0.14, 5.46)	0.0805	7.23 (1.83, 12.62)	0.00128	1.46 (0.15, 2.77)	0.00016
No	−0.18 (−2.22, 1.86)	1.00	7.03 (5.12, 8.93)	<0.00001	15.84 (12.17, 19.50)	<0.00001	5.10 (4.21, 5.99)	<0.00001
Other								
Yes	−0.21 (−2.28, 1.87)	1.00	3.94 (2.06, 5.81)	<0.00001	10.21 (6.59, 13.82)	<0.00001	2.82 (1.94, 3.72)	<0.00001
No	−0.16 (−3.01, 2.68)	1.00	8.86 (6.29, 11.43)	<0.00001	18.60 (13.65, 23.55)	<0.00001	6.07 (4.85, 7.29)	<0.00001

^a^ yes’ indicates that patients were diagnosed with this type of condition yet could have diagnoses of other conditions; ‘no’ indicates that patients were not diagnosed with this type of condition yet could have had diagnoses of other conditions. bpm, beats per minute; ms, milliseconds.

### 3.1 General findings

For the three, time-domain HRV metrics, there were significantly less agreement between ECG (chest and bicep) and PPG based metrics (see Figure 1; Supplementary Table S2). The PPG-derived PRV consistently underestimated rMSSD, SDNN, and pNN50 when compared to ECG-Chest and ECG-Bicep. For rMSSD, statistically significant differences were found for ECG-Bicep vs. PPG-Bicep (mean difference = −5.6 ms, 95% CI: −4.2, −7.1, p < 0.00001). The comparison between ECG-Chest and PPG-Bicep exhibited a nearly identical statistical difference (mean difference = −5.6 ms, 95% CI: 4.2, 7.0). Similarly, the Pearson correlation coefficients were smaller between the ECG and PPG measures for rMSSD (r = 0.99 vs. 0.75). Like rMSSD, the degree of agreement between ECG and PPG measurement methods for SDNN was wide. PPG-Bicep significantly underestimated SDNN in both the ECG-Bicep vs. PPG-Bicep (mean difference: −13.1 ms, 95% CI: −10.3, −15.9, p < 0.00001) and ECG-Chest vs. PPG-Bicep (mean difference: −13.1 ms, 95% CI: −10.3, −15.9, p < 0.00001). The PPG-Bicep also exhibited a lower Pearson correlation coefficient compared to the ECG-Chest (r = 0.99 vs. 0.84). The same differences were observed for pNN50, where the PPG-Bicep showed lower agreement with ECG-Chest and ECG-Bicep (mean difference: −3.9 ms, 95% CI: −3.3, −4.6, p < 0.00001). Additionally, the Pearson correlation coefficient was the lowest, indicating poorer agreement for pNN50 with the ECG-Chest (r = 0.99 vs. 0.67).

#### 3.1.1 Stratified results

The mean differences in HRV between PPG-Bicep and ECG-Bicep across 5 chronic disease categories are noted in Table 2.

The significant mean differences in rMSSD were as follows: *cardiovascular:* 3.04 ms, 95% CI: 1.33, 4.75, p < 0.00001, *endocrine*: 2.85 ms, 95% CI: 0.52, 5.18, p < 0.00001, *neurological:* 4.39 ms, 95% CI: 1.39, 7.39, p = 0.00027, and *other:* 3.94 ms, 95% CI: 2.06, 5.81, p < 0.00001. No significant differences in rMSSD were observed for the *respiratory* category: 2.66 ms, 95% CI: −0.14, 5.46, p = 0.0805.

For SDNN, significant differences between ECG-Bicep and PPG-Bicep were observed for *cardiovascular:* 8.50 ms, 95% CI: 5.25, 11.74, p < 0.00001, *endocrine:* 8.43 ms, 95% CI: 3.97, 12.90, p < 0.00001:, *neurological:* 11.84 ms, 95% CI: 6.02, 17.67, p < 0.00001, *respiratory:* 7.23 ms, 95% CI: 1.83, 12.62, p = 0.00128, and *other:* 10.21 ms, 95% CI: 6.60, 13.82, p < 0.00001.

For pNN50, significant differences between ECG-Bicep and PPG-Bicep were observed for *cardiovascular:* 2.48%, 95% CI: 1.67, 3.3, p < 0.000018, *endocrine*: 2.21% 95% CI: 1.12, 3.29, p < 0.00001, *neurological:* 2.91%, 95% CI: 1.25, 4.32, p < 0.00001 *respiratory:* 1.46%, 95% CI: 0.15, 2.77, p = 0.00016, and *other:* 2.82%, 95% CI: 1.94, 3.72, p < 0.00001).

#### 3.1.2 Graphical interpretations

**FIGURE 3:**
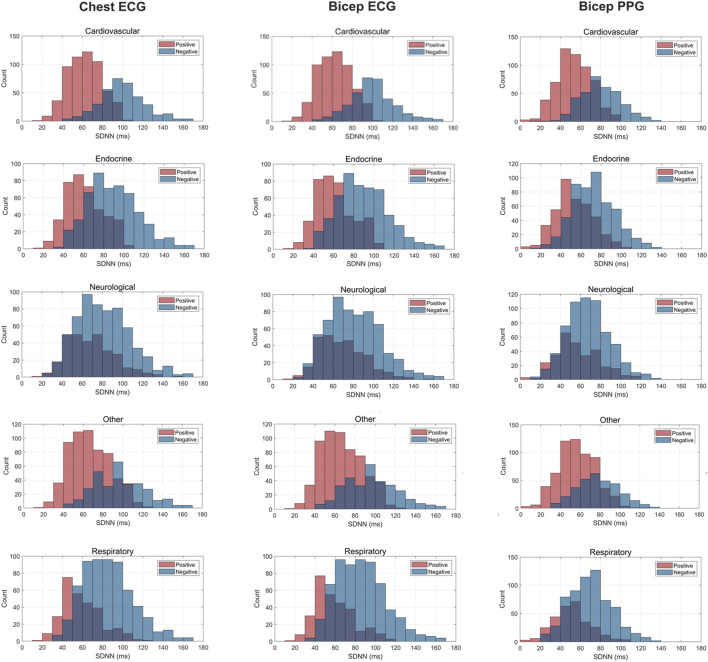
Histogram Plots for SDNN (ms) Measured by ECG and PPG Across Several Common Chronic Diseases. ECG location–chest and bicep. PPG location–bicep.

**FIGURE 4:**
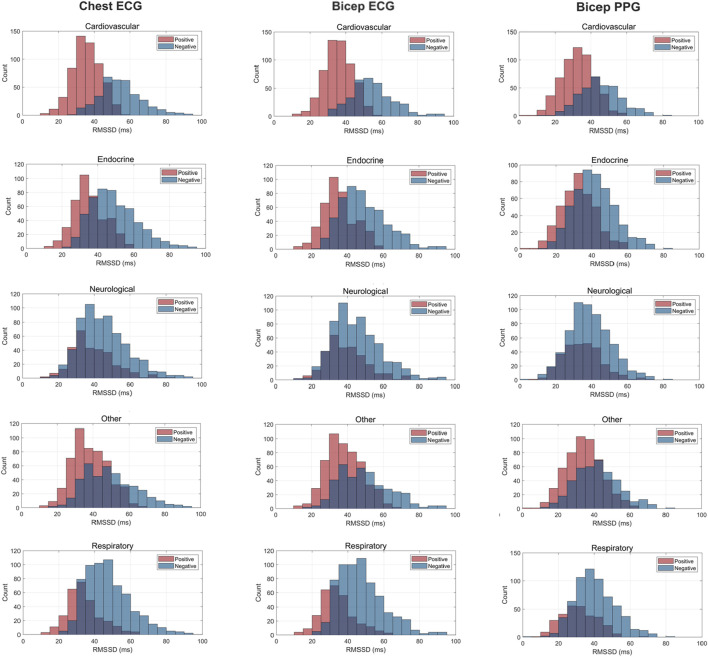
Histogram Plots for rMSSD (ms) Measured by ECG and PPG Across Several Common Chronic Diseases. ECG location–chest and bicep. PPG location–bicep.

**FIGURE 5:**
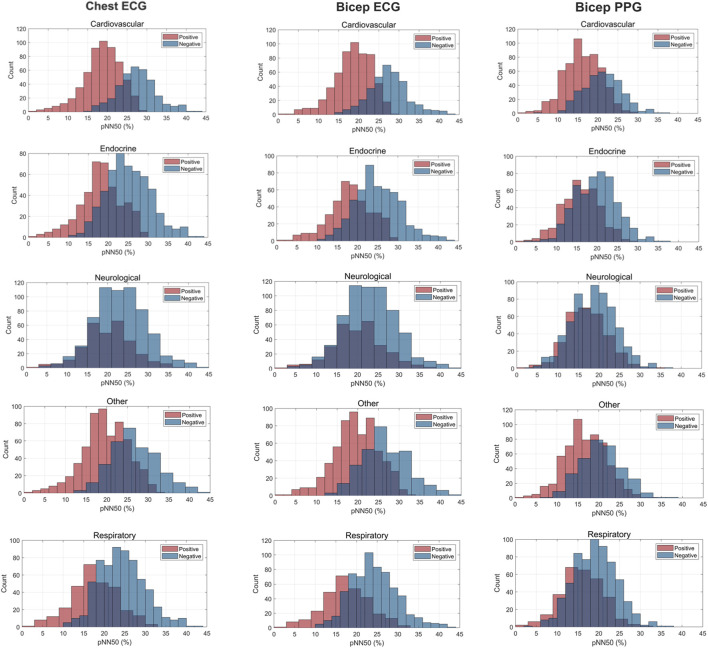
Histogram Plots for pNN50 (%) Measured by ECG and PPG Across Several Common Chronic Diseases. ECG location–chest and bicep. PPG location–bicep.

Figures 3–5 depict the comparisons in the distributions for each chronic disease category for rMSSD, SDNN and pNN50. Across all conditions, the distributions for all HRV metrics derived from PPG-Bicep were narrower and exhibited a leftward shift.

## 4 Discussion

This large-scale, unique study examined the agreement between PR/PRV and HR/HRV in a beat-to-beat analysis using a diverse sample of adults. The major findings of our study were 1) for the total sample, PRV metrics measured by PPG exhibited poor agreement with all HRV metrics measured by ECG at the chest and bicep locations, 2) across all major chronic conditions evaluated, statistically significant differences between PRV and HRV were observed with PRV consistently underestimating rMSSD, SDNN and pNN50, 3) marked differences were observed between the ECG (electrical) and PPG (fluid) waveforms and, 4) no statistically significant differences were found between PR and HR values for ECG and PPG methodologies. Here, PRV significantly underestimated HRV, rendering it an unacceptable surrogate that is strongly attributed to the striking differences in their respective methodologies.

**FIGURE 6:**
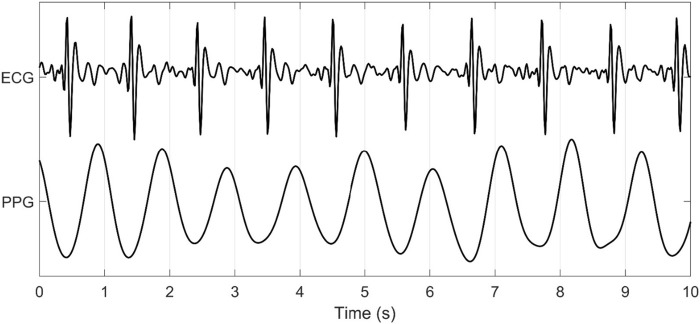
ECG and PPG Signal Output from the Warfighter Monitor™ over a 10-s interval.

In the current study, PRV consistently demonstrated poor agreement with all HRV metrics evaluated. Specifically, PRV exhibited lower values compared to HRV, showing a bias towards underestimation. This observation is rather intuitive given the marked dissimilarities in PPG and ECG methodologies. PPG does not measure the electrical activity of the heart like an ECG (Mejía-Mejía et al., 2020a; Mejía-Mejía et al., 2020b), but rather the blood volume changes occurring following each phase of the cardiac cycle, presenting two significant issues. First, several factors influence blood volume independently of cardiac activity, such as arterial stiffness and blood pressure. These factors can introduce substantial changes in signal morphology (Fine et al., 2021; Pi et al., 2021; Rovas et al., 2023), which in turn could lead to differences in PRV metrics, ultimately weakening their correlation with HRV. Further, higher amounts of melanin (Bermond et al., 2023) and subcutaneous adipose tissue distort the scattering of light, affecting the amount of light penetrating the skin and intensity of light absorbed (Ajmal et al., 2021). Additionally, tattoos, which introduce pigments and scarring, can further interfere with light scattering and absorption, potentially distorting the PPG signal and affecting the accuracy of measurements (Scardulla et al., 2023). Second, the waveforms generated from PPG signals are in stark contrast to the QRS complex derived from ECG (see Figure 6). The structure of the vasculature naturally creates a low-pass filter. This reduces high-frequency signals resulting in a waveform with smooth, rounded peaks. Consequently, identifying fiducial points becomes increasingly difficult with the highest amplitude of each pulse wave generated assumed synonymous to the R peak. Further, a lack of synchronicity in the PPG waves and ECG-QRS complexes exists (Figure 6), likely due to the inertial resistance in the vasculature affecting the acceleration and deceleration of blood volume changes between the systolic and diastolic phases of the cardiac cycle.

The multitude of factors affecting PPG significantly reduces its granularity, affecting its accuracy in measuring HRV. Figures 3–5 effectively demonstrate the decreased precision of PPG and its derivatives with narrower distributions observed across all chronic conditions evaluated. This phenomenon equivalently occurred in both males and females. Detecting the subtle variations between heartbeats is paramount in ascertaining critical information on ANS activity and function. Although PRV and HRV both showed lower values for rMSSD, SDNN and pNN50 among patients exhibiting a chronic condition, a larger leftward shift was observed for the cardiovascular, endocrine, neurological, respiratory and “other” distributions. Many studies previously demonstrated unhealthy patients often present with lower HRV patterns indicating abnormal ANS activity (Hu et al., 2018; Trivedi et al., 2019; Escutia-Reyes et al., 2021; Wu et al., 2021). However, as shown here, the sensitivity of PRV to abnormalities in ANS activity is lower than that of HRV, which could critically impact clinicians’ ability to accurately assess the type and severity of a condition, ultimately influencing treatment decisions. As such, for patients to experience improved health outcomes, clinicians must utilize a highly precise methodology for measuring HRV that captures both the direction and magnitude of its patterns.

Our study uniquely utilized ECG and PPG from a single device. The ECG-Bicep measure reached a near-perfect correlation for all HRV metrics and showed no significant differences with the ECG-Chest measure, highlighting two important points. First, the poorer performance of PPG in measuring HRV compared to the ECG cannot be attributed to differences in measurement site, as previous studies have consistently suggested. In the current study, the poor agreement and significant differences observed between PRV and HRV were nearly identical at the chest and bicep locations and independent of PPG wavelength (red, green and infrared). Second, ECG technology can be utilized in non-clinical settings and with non-invasive, wearable devices, like the WFM used in the current study. While PPG technology does not provide accurate HRV it does provide significant value in measuring other health-related metrics. For instance, PPG is used for measuring blood oxygen saturation levels and changes in blood properties (e.g., clotting, PPG dropouts), etc., In clinical healthcare settings (Schultz-Ehrenburg and Blazek, 2001). Thus, utilizing PPG as a standalone methodology for measuring these other health metrics in conjunction with ECG-derived HRV, provides a more accurate and comprehensive health profile (Yuda et al., 2020a).

While former studies documented similar significant differences between PRV and HRV values, authors often concluded that PPG was a “reasonable”, non-invasive alternative (Kiran Kumar et al., 2021). For example, Sarhaddi et al., 2022, investigated the validity of a Samsung smartwatch (PPG device) and showed moderate correlations with ECG (SDNN: 0.80, rMSSD: 0.78, Low-Frequency (LF): 0.78, high-frequency (HF): 0.78 and LF/HF: 0.62), yet concluded the PPG provided “acceptable” values. Another study by Cao et al. (2022) evaluating the Oura Ring, showed poor-to-moderate correlations with six out of seven HRV indices (0.35–0.82), yet concluded the Oura Ring provided “acceptable” levels of validity. Similar reports were found in a study by Natarajan et al. (2020a) which investigated HRV with PPG using data derived from FitBit wearable devices in a sample of 8 million people. The most striking concern of this study, and many others using commercial devices (Natarajan et al., 2020b; Gupta et al., 2023), is the widespread replacement of PRV with HRV, which occurs not only in scientific studies published in top-tier, peer-reviewed journals (e.g., the Lancet, Nature Publishing Group) but also marketed as such by many companies manufacturing wearable devices (de Vries et al., 2023, Gaur et al., 2024, Jasinski et al., 2024, Wyatt et al., 2020. Perhaps, in some circumstances, “reasonable” is “good enough”. However, in the context of healthcare, “good enough” is unacceptable and dangerous.

### 4.1 Consequences and implications for clinical application

Healthcare agencies are rapidly integrating AI for improving patient outcomes, interpreting diagnostic testing and tracking health metrics (Secinaro et al., 2021; Alowais et al., 2023). To accomplish this, AI analyzes large amounts of patient data to detect patterns and relationships of varying health-related outcomes. Critically, algorithm prediction and decision accuracy entirely depend on the quality of training and test data (Akinrinmade et al., 2023). Thus, using inaccurate and improperly labelled HRV data derived from PPG to train AI models will result in poor outcomes (“garbage in = garbage out”). Examples of the significant health-related consequences include misidentifying systemic physiological changes indicative of disease, leading to a misdiagnosis, poor tracking of disease progression, and selection of ineffective treatments. Moreover, non-representative AI models may further widen the existing health disparities observed among minority race/ethnic groups (Arora et al., 2023). Given this, using properly labeled HRV data only derived from highly accurate methodologies (i.e., ECG) could not be more imminent and necessary (Challen et al., 2019).

Until the relevant scientific and clinical communities recognize, accept and establish ECG-HRV and PPG-PRV as distinct, standalone measures of ANS activity, clinical recommendations for using and interpreting data collected via wearable devices will remain undeveloped. The findings from the current study call to action the need for clinicians to exercise caution when interpreting PPG-PRV data by understanding its fundamental differences from HRV. Further, if clinicians continue collecting PPG data, utilizing it as a marker of ANS activity, it is imperative to label and interpret it as PRV and a separate metric from HRV.

### 4.2 Strengths and limitations

This study has several strengths. First, we conducted one of the largest and most diverse studies measuring both ECG and PPG. Our sample included 931 individuals exhibiting diverse demographic and health profiles, which likely better represents the United States adult population as nearly 42% of United States adults present with at least one chronic disease morbidity. Previous studies often included only young, healthy individuals, thereby significantly reducing the generalizability of their findings. Second, the ECG and PPG methods measured HRV from the same device and location, controlling for any differences possibly attributed to these factors. Third, we included an additional ECG measurement location (at the chest), to validate the differences in ECG and PPG observed at the bicep location. This study also has some limitations. First, our study employed a cross-sectional design limiting conclusions on longitudinal findings. Second, our study only included adults and thus, we cannot generalize our findings to individuals under the age of 18 years who likely exhibit different physiological responses affecting PRV and HRV. Third, factors influencing HRV such as medication use, activity levels, sleep quality, etc., were not measured, potentially influencing the interpretations of our study findings. Lastly, other markers of ANS function exist like blood pressure, cardiac output, vascular resistance, etc., all of which were not included, precluding a comprehensive assessment of ANS function.

### 4.3 Conclusion

Our study demonstrated significant disagreement between PRV and HRV derived from PPG and ECG methodologies, respectively. PPG-based PRV underestimated all ECG-based HRV metrics at both the bicep and chest measurement sites and across several chronic diseases exhibited by a large proportion of US adults. Importantly, PPG appeared to non-uniformly underestimate HRV, eliminating the possibility of utilizing a correction factor. While PPG accurately measures other health metrics, it serves as an invalid surrogate for HRV, greatly attributed to its demonstrable differences in methodology relative to ECG. We strongly recommend that future investigations employ longitudinal studies to track changes in ECG-HRV and PPG-PRV, evaluate the influences of demographics like age, race, ethnicity, sex, etc., and incorporate other metrics of ANS activity to explore deeper insights and causal relationships.

## Data Availability

The raw data supporting the conclusions of this article will be made available by the authors, without undue reservation.
