# Supplementary material for: Pulse rate variability is not the same as heart rate variability: findings from a large, diverse clinical population study
Source: Front Physiol. 2025 Jul 30;16:1630032. doi: 10.3389/fphys.2025.1630032 (PMC12343505; doi:10.3389/fphys.2025.1630032)
Supplement: Supplementary file 1 [file Table1.docx]

| **Health Condition** | **# of Conditions** | **Prevalence** | **Major Contributing Conditions** |
| --- | --- | --- | --- |
| *Respiratory* | 13 | 31.6% | COPD (16.9%), Emphysema (17.2%), Smoker (17.6%), and Asthma (18.2%) |
| *Metabolic* | 5 | 58.8% | Obesity (47.8%), Dyslipidemia (25.7%) and Type 2 Diabetes Mellitus (26.2%) |
| *Cardiovascular* | 20 | 61.2% | Anemia (11.8%), Dysrhythmias (23.9%) and Hypertension (29.7%) |
| *Mental Health* | 7 | 38.6% | Anxiety (38.6%) and Depression (27.3%) |
| *Gastrointestinal* | 15 | 27.9% | Peptic Ulcer Disease (27.9%) and GERD (27.9%) |
| *Neurological* | 18 | 8.8% | Alzheimer’s & Dementia (8.8%), CVA (5.1%), Headaches & Migraines (4.4%) |
| *Hormonal* | 8 | 23.7% | Thyroid Disease (23.7%) |
| *Renal* | 5 | 6.3% | Chronic Renal Insufficiency/Disease (6.3%) and Nephrolithiasis (2.7%) |
| *Skeletal* | 8 | 1.5% | Osteoarthritis (1.5%) and Gout (1.3%) |
| *Immune* | 8 | 2.1% | HIV/AIDS (2.1%) and Rheumatoid Arthritis (0.8%) |

**Table S1**. Prevalence of Health Conditions, Extracted from Medical Records, Among Study Sample.

COPD = Chronic Obstructive Pulmonary Disease; GERD = Gastroesophageal Reflux Disease; HIV = Human Immunodeficiency Virus; AIDS = Acquired Immunodeficiency Syndrome.
