# Supplementary material for: Pulse rate variability is not the same as heart rate variability: findings from a large, diverse clinical population study
Source: Front Physiol. 2025 Jul 30;16:1630032. doi: 10.3389/fphys.2025.1630032 (PMC12343505; doi:10.3389/fphys.2025.1630032)
Supplement: Supplementary file 2 [file Table2.docx]

**Table S2**. Mean differences in ANS Marker Estimates by Measurement Method

*denotes statistical significance at p<0.00001

|  | **Measurement Method of ANS Function** | | |
| --- | --- | --- | --- |
|  | *ECG-Bicep vs ECG-Chest* | *ECG-Bicep vs PPG-Bicep* | *ECG-Chest vs PPG-Bicep* |
| **ANS Marker** | **Mean Difference**  **(95% CI)** | **Mean Difference**  **(95% CI)** | **Mean Difference**  **(95% CI)** |
| *Heart Rate (bpm)* | 0.05 (-1.5, 1.6) | -0.2 (-1.8, 1.4) | -0.2 (-1.8, 1.4) |
| *rMSSD (ms)* | 0.04 (-1.3, 1.5) | 5.6 (4.2, 7.1)* | 5.6 (4.2, 7.0)* |
| *SDNN (ms)* | -0.01 (-2.8, 2.8) | 13.1 (10.3, 15.9)* | 13.1 (10.3, 15.9)* |
| *pNN50 (ms)* | -0.02 (-0.7, 0.6) | 3.9 (3.3, 4.6)* | 3.9 (3.3, 4.6)* |
